# The Systemic–Evolutionary Theory of the Origin of Cancer (SETOC): A New Interpretative Model of Cancer as a Complex Biological System

**DOI:** 10.3390/ijms20194885

**Published:** 2019-10-02

**Authors:** Antonio Mazzocca

**Affiliations:** Interdisciplinary Department of Medicine, University of Bari School of Medicine, Piazza G. Cesare, 11, 70124 Bari, Italy; antonio.mazzocca@uniba.it; Tel.: +39-080-5593-593

**Keywords:** cancer theories, endosymbiosis, evolution, complex systems, emerging properties, atavism

## Abstract

The Systemic–Evolutionary Theory of Cancer (SETOC) is a recently proposed theory based on two important concepts: (i) Evolution, understood as a process of cooperation and symbiosis (Margulian-like), and (ii) The system, in terms of the integration of the various cellular components, so that the whole is greater than the sum of the parts, as in any complex system. The SETOC posits that cancer is generated by the de-emergence of the “eukaryotic cell system” and by the re-emergence of cellular subsystems such as archaea-like (genetic information) and/or prokaryotic-like (mitochondria) subsystems, featuring uncoordinated behaviors. One of the consequences is a sort of “cellular regression” towards ancestral or atavistic biological functions or behaviors similar to those of protists or unicellular organisms in general. This de-emergence is caused by the progressive breakdown of the endosymbiotic cellular subsystem integration (mainly, information = nucleus and energy = mitochondria) as a consequence of long-term injuries. Known cancer-promoting factors, including inflammation, chronic fibrosis, and chronic degenerative processes, cause prolonged damage that leads to the breakdown or failure of this form of integration/endosymbiosis. In normal cells, the cellular “subsystems” must be fully integrated in order to maintain the differentiated state, and this integration is ensured by a constant energy intake. In contrast, when organ or tissue damage occurs, the constant energy intake declines, leading, over time, to energy shortage, failure of endosymbiosis, and the de-differentiated state observed in dysplasia and cancer.

## 1. Introduction

**Figure 1:**
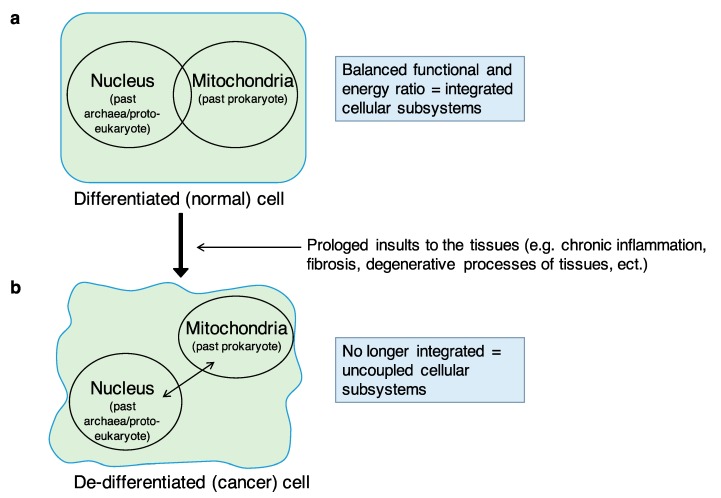
The balanced integration of the two endosymbiotic subsystems, (i) information = nucleus (the ancestral ‘‘archaea”) and (ii) energy = mitochondria (the ancestral ‘‘prokaryote”) within the eukaryotic cell is essential to maintain the differentiated cell status. The transition from (**a**) the differentiated (normal) cell to (**b**) the de-differentiated (cancer) cell as a consequence of a prolonged insult to the tissues (e.g., chronic inflammation, fibrosis, degenerative processes of tissues, etc.) could be caused by the progressive breakdown (or uncoupling) of the two endosymbiotic subsystems integration. The resulting de-emergence of the “eukaryotic cell system” with the re-emergence of the archaea-like and prokaryotic-like subsystems, featuring uncoordinated behaviors, would lead to a sort of “cellular regression” towards ancestral or atavistic biological functions and behaviors similar to those of protists or unicellular organisms in general.

I recently proposed the systemic–evolutionary theory of cancer (SETOC) as a new theory about the origin of cancer [1,2,3]. The SETOC postulates that one of the fundamental processes underlying the origin of cancer is the progressive breakdown or uncoupling of endosymbiosis between two cellular “subsystems”, namely, (i) the mitochondria/cell bioenergetics (the ancestral prokaryote) and (ii) the informational nuclear–cytoplasmic component (the ancestral archaea), which have co-evolved into the eukaryotic cell. The concept of endosymbiosis here mostly refers to the symbiogenesis or endosymbiotic theory according to Lynn Margulis. Lynn Margulis was an American biologist who theorized endosymbiosis and the idea that evolution is strongly based on cooperation [4,5]. Factors causing the breakdown (or the uncoupling) of endosymbiosis include all those factors that induce, over time, persistent alterations of tissues or tissue damage including cyto-architectural degenerations, which activate processes such as chronic inflammation and repair processes as well as fibrogenesis, along with the genetic/epigenetic modifications and vascular/stromal changes observed in cancer. In this perspective, passing through chronic inflammation and the various degrees of dysplasia, cancer can be seen as an attempt or extreme response to the impossibility of removing the causal factors of tissue damage. Thus, the SETOC proposes that, as a consequence of long-term injuries, the progressive uncoupling of the endosymbiotic subsystems—which are entirely integrated under physiological conditions—generates alterations in cellular organization (lower level) and in tissue interactions (upper level) that are important driving forces of neoplastic transformation (Figure 1).

In the systemic view of the SETOC, emerging properties arising at the cellular level from the uncoupling of the integrated endosymbiotic subsystems affect tissue alterations at the organizational level within the tissue. In turn, tissue alterations perturb cellular levels by causing the uncoupling of endosymbiosis, since a tissue cannot exist without cellular integration in metazoans. In fact, in this view, the disruption of tissue architecture can itself be a factor generating the breakdown of the endosymbiotic cellular system integration. It is well accepted that a normal tissue homeostasis and preserved tissue architecture prevent cancer development and progression [6]. If cells are not constrained or fully integrated in the structural and functional organization of the tissue, they can exhibit unicellular organism-like characteristics and sometimes primordial behaviors such as those often observed in cancer cells. This supports the concept that insults to a tissue, by affecting its architecture, can cause the uncoupling of cellular endosymbiosis. Just like the parallelism of microcosm and macrocosm, the endosymbiotic integration of mitochondria within eukaryotic cells somehow evokes the subcellular connections between microbiome and macroorganisms. In the latter case, there is now evidence that microbes co-living with organisms may confer susceptibility to develop some forms of cancers and may also influence the response to therapy [7].

Biological tissues can be thought of as “self-organized” structures which become ordered in space and in time through emerging properties deriving from the proper integration and function of eukaryotic cells [8,9]. Cancer, as an entity developing from a tissue, could therefore be considered as “an emerging property developing in another emerging property”, namely, the “tissue”, or alternatively, a “complex system arising from another (pre-existing) complex system” (the tissue), but with changed patterns of self-organization. As mentioned above, the breakdown of the cooperation of cellular subsystems can be caused by a wide array of causes including alterations and impairments that inevitably impact tissue organization, such as alterations of the tissue microenvironment (mainly due to processes such as chronic inflammation, fibrosis, etc.), damage to the nuclear and/or mitochondrial genomes (e.g., by viruses, chemicals, several agents including foodborne agents), which control metabolic pathways, mitochondrial energy production, etc.; all of these are risk factors known to favor the onset of cancer. Thus, the uncoupling of two evolutionarily conserved subsystems in eukaryotic cells drives them towards neoplastic transformation. Importantly, the two “subsystems” are fully integrated in normal conditions, and this integration is ensured by a constant energy intake, necessary to maintain the differentiated state of the cell. Cellular differentiation is therefore an “emerging” function. The constant energy intake declines when organ or tissue damage occurs. In fact, after a prolonged insult or organ damage (i.e., chronic inflammation, fibrosis, degenerative processes of tissues), a reduced supply of oxygen and nutrients ensues, and therefore, a reduction of energy at the cellular level, following which, the two subsystems may undergo a gradual process of uncoupling. In other words, when enduring insults compromise a tissue organization, the amount of energy needed to maintain cell differentiation becomes limited, and this causes, over time, a state of de-differentiation or pre-neoplastic condition. In addition to energy problems, the failure of endosymbiosis can cause defects in cell division and chromosomal abnormalities in terms of both number (i.e., aneuploidy) and structure (e.g., deletions, duplications, translocations, etc.). Energy problems and defects in cell division are obviously interconnected, being two sides of the same coin. The biological systems, tissues included, are systems characterized by “robustness”, which is the ability to maintain performance under a wide range of perturbations or conditions of uncertainty [10]. Despite this robustness, the biological systems can display fragility with respect to certain types of perturbations or particular insults. The progressive rupture or failure of endosymbiosis as a consequence of long-lasting insults or particular insults, for example, could compromise and weaken this key property of the biological systems.

The SETOC also posits that the de-emergence of the “eukaryotic cell system” and re-emergence of the archaea-like and prokaryotic-like subsystems, featuring uncoordinated behaviors leading to cellular transformation, result in a sort of “cellular regression” towards ancestral or atavistic biological functions and behaviors (similar to those of protists or unicellular organisms in general). Cancer cells resemble unicellular organisms (e.g., prokaryotes or amoebas) in certain biological behaviors, such as those related to their proliferative and metabolic properties, invasiveness, and the capability to activate the immune response. Primordial or atavistic behaviors are undoubtedly not displayed by eukaryotic cells in normal conditions but are preserved by cells as a phylogenetic “memory”. This memory could re-emerge during neoplastic transformation. In addition to the phylogenetic memory, an ontogenesis-linked memory can also emerge in neoplastic cells that often exhibit embryonic-like properties. Regarding cellular functions and the evolutionary throwback in cancer, we can assume that the mitochondrion has “learned” respiration during its co-symbiotic evolutionary process in eukaryotic cells, without phylogenetically forgetting the ancestral function of fermentation (typical of most prokaryotes or certain unicellular organisms). This, for example, is one of the functions that generally “resurface” in cancer cells. It is like the operating system of a latest generation computer, which uses the latest version for which it was designed but, “inside”, it retains all the previous versions on which this operating system was developed. This is not simply a metaphor but a new interpretative model that may offer a useful pathogenic and pharmacological basis for new therapeutic approaches to cancer. To this end, a paradigm shift [11] in cancer pharmacology is needed, since our knowledge of the pathophysiology of cancer (as a complex system) is still insufficient, despite a wide knowledge of the genetic and molecular mechanisms (reductionist approach). In fact, the current cancer pharmacology deals only with microvariables (i.e., genetic or protein targets which do not take into account the integrated pathophysiology of cancer) rather than macrovariables (for example, membrane potentials, electromagnetic fields, cellular communications, etc.) or mesovariables (between micro- and macrovariables, such as the interaction between various cellular components including organelles). This paradigm shift could allow cancer pharmacology to move forward, beyond the current “molecular” treatments, mainly focused on single targets (e.g., targeted therapy). Alternatively, we should consider “modular” treatments that target cancer-associated processes (such as inflammation, coagulation, etc.) or even forms of “eco-system” treatments that address the whole functioning of the cancer ecosystem (comprising the microenvironment where the tumor arises and develops in the context of the whole organism) [12,13]. An example of ecosystem treatment could be inspired by the so-called “biomimicry” that is a way of emulating or mimicking what nature does, for example, in plants, through the use of blends of natural compounds that plants produce to defend themselves from their aggressors (molds, lichens, bacteria, fungi, arthropods, herbivores, etc.). Another example could be the use of variable electromagnetic fields acting on particular biological processes in tumor cells. In other words, new theoretical working models are needed on which to base new anti-tumor therapeutic approaches. The SETOC will hopefully meet this need.

## 2. Further Explanations

**Figure 2:**
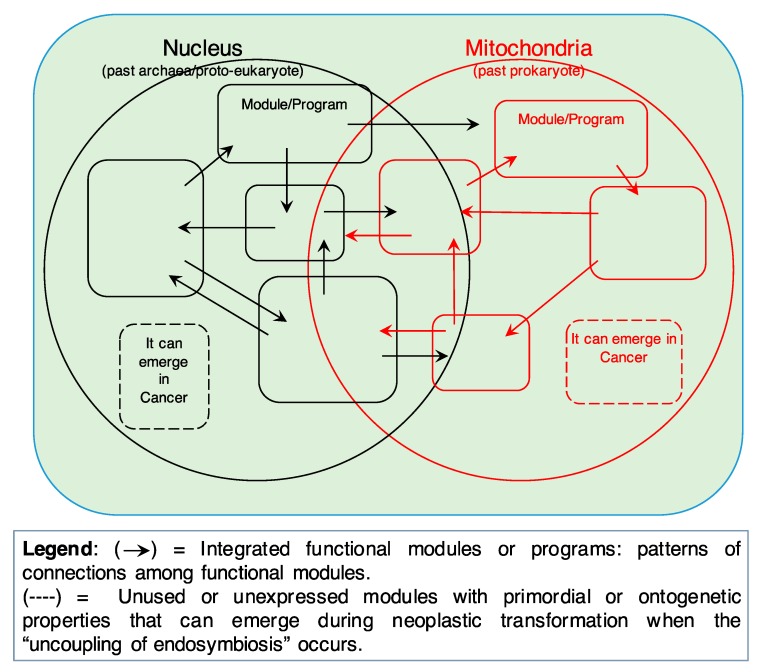
Both informational (past archaea/proto-eukaryote) and energy/mitochondrial (past prokaryote) components should be viewed as made up of fully functional integrated modules or programs. Modules integrate information according to patterns of connections (→) and are highly flexible. In fact, different modules can share a given component depending on the functional status of the cell (robustness of cellular networks). However, unused or unexpressed modules (dashed lines) with primordial/unicellular-like properties (or reminiscences of these properties in the functionally integrated modules) can be present in eukaryotic cells, and “emerge” during neoplastic transformation when the “uncoupling of endosymbiosis” occurs. This would confer primordial/unicellular-like properties to cancer cells (i.e., fermentative glycolysis, adaptation to anaerobicity, cell movement, etc.). These properties may reside, as an archaic memory, even in the cellular modules in current use (solid lines), unexpressed under normal conditions but re-expressed by cancer cells.

The prevalent theory tries to explain the origin of cancer by positing DNA mutations as the primary cause/driver of cancer formation and progression. Although it is important here to remember that the term “cancer” refers to a galaxy of diseases that are very different from one another as regards their genesis, causal factors, and evolution, nevertheless, they share basic properties such as autonomous and uncontrollable proliferation and disengaging from the hierarchical levels that regulate multicellular organisms. The SETOC, while recognizing the presence of DNA mutations in different types of cancer, offers a different view according to which a primary importance is attached to: (i) evolution (Margulian [4,5], understood as cooperation and symbiosis) and (ii) integration into a real system of fundamental cellular components, as expected in any complex system. More precisely, this new theory focuses on cancer as a complex entity that, in turn, develops from an equally complex system like a biological tissue, which steadily undergoes changes in organ geometry and boundary constraints, biomechanical and biophysical laws, biochemical and microenvironmental influences, and so on. In addition, a complex system relies on the fact that specialized functions in eukaryotic cells are organized or carried out by programs or “modules” [14]. The SETOC assumes that cells also harbor within themselves unexpressed or partially expressed archaic programs or modules, which are the result (or the “residue”) of a complex evolutionary process (Figure 2). 

**Figure 3:**
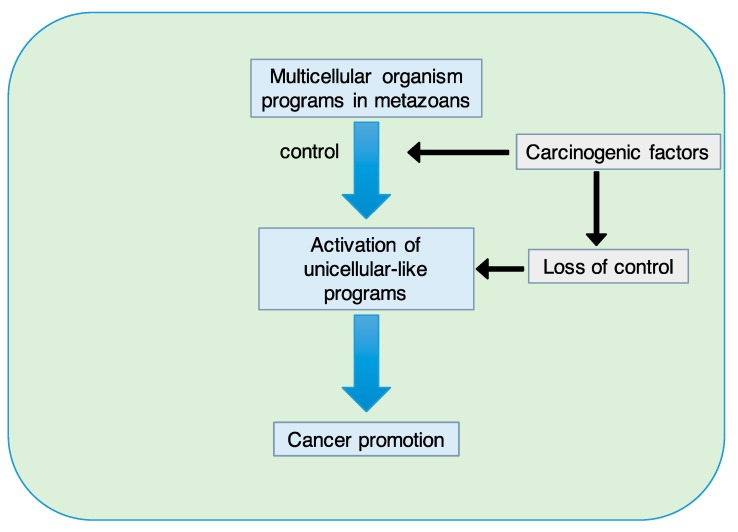
When properly functioning, multicellular programs are believed to be oncosuppressive. The loss of control over these programs caused by cancer-promoting factors may result in the activation of unicellular-like programs that promote cancer.

These processes can resurface (or re-emerge) in pathological conditions such as cancer. In this context, the SETOC contemplates the fact that multicellular programs, when properly functioning, are somehow oncosuppressive per se. The loss of control over these programs, caused for example by carcinogenic factors, can activate unicellular-like programs to promote cancer (Figure 3). 

The SETOC is an attempt to model these complex systems on the basis of the concept of two subsystems, evolutionarily conserved in eukaryotic cells, namely, the energy subsystem represented by the mitochondria (the archaic prokaryote) and the informational subsystem represented by the nucleus–cytoplasm (the archaic archaea or even the proto-eukaryotic cell). These two subsystems, perfectly integrated in the “modern” eukaryotic cell, have co-evolved to the present day, giving cells the characteristics we normally observe (i.e., specialized or differentiated cells). The progressive “breakdown” or the failure of this form of integrated endosymbiosis between the two subsystems, resulting, for example, from prolonged tissue insults or organ damage, could lead to a gradual process of “decoupling” of the two subsystems, affecting the cellular organization in a tissue (cellular de-differentiation) and driving it towards neoplastic transformation. There is evidence supporting constituent elements of the theory presented here at the cellular and molecular levels, particularly with regard to the interplay between mitochondria and nucleus. For example, a number of studies show that when the communication between mitochondria and the nucleus fails, mitochondria become dysfunctional, and tumorigenesis can be triggered [15,16,17,18]. This evidence is emphasized by the importance of mito–nuclear communications in human cancer, as demonstrated by recent findings showing an increased somatic transfer of mitochondrial DNA (mtDNA) in colorectal tumors [19].

The cellular de-differentiation associated with the breakdown of the mito–nuclear system is mutually linked to a sort of “de-emergence” which takes place within the “eukaryotic cell system”, with the consequent re-emergence of the “atavistic” bacterial–prokaryotic subsystem or the primordial archaea–cell subsystem, each one featuring a self-contained, uncoordinated biological behavior. This would result in a sort of “cellular regression” of cancer cells to primordial or atavistic biological functions and behaviors similar to those of bacteria or unicellular organisms in general. However, the concept of unicellular-like properties must be put in the right context, because cancer cells often grow as colonies exhibiting a certain cooperativity. Cancer cells can, for example, migrate and form metastases as single cells or as groups of cells. Indeed, these are biological behaviors closely resembling the bacterial behavior, such as the growth of bacterial colonies or bacteria whole-body dissemination (bacteremia–sepsis). Thus, cancer cells display behavioral properties similar to those of unicellular organisms, especially those with colonial capabilities. 

These properties are possessed in nature by certain organisms such as protists (e.g., slime molds). The SETOC, based on the re-emergence of ancestrally conserved subsystems (similar to unicellular organisms such as protists, prokaryotes, etc.), suggests the potential of pharmacological anti-cancer strategies similar to those adopted by plants in nature to oppose the aggression by microorganisms (“biomimicry”, that is, the action of emulating or mimicking models, systems, and elements of nature). In this regard, plants provide an excellent example. Many plants use blends or cocktails of natural compounds that they produce and release in order to neutralize attacking microorganisms (e.g., molds, lichens, bacteria, fungi, etc.). Obviously, the strategy is based on the use of “blends” of substances, sometimes containing numerous anti-bacterial or anti-fungal compounds against which it is difficult to develop an adequate resistance because of the large number of compounds they contain (generally, bacterial resistance can be developed for one or very few antibiotics). Similar strategies could be designed to counteract cancer cells (difficult to halt with a single drug or the association of few drugs), which display metabolic, proliferative, and invasive characteristics similar to those of unicellular organisms. Therefore, potential benefits of integrating effective blends of natural compounds into anticancer therapies or other beneficial practices such as fasting, which may reset cellular functions, are probably worth mentioning here [20,21]. The SETOC offers some ideas and bases for a paradigm shift [11] in cancer pharmacology. Indeed, a critical but effective conceptual step forward has recently been taken in this direction [22].

## 3. Conclusive Remarks

The concepts presented here are an attempt to frame the complexity of cancer and, if possible, to provide elements supporting a new approach to more effective therapeutic interventions against cancer. The SETOC is, indeed, a new interpretative model that helps to view cancer as an integrated and complex system and it also offers a wider basis on which to design anti-cancer strategies as well as a more comprehensive cancer pharmacology. In this regard, a paradigm shift in cancer pharmacology is needed, since our knowledge of the pathophysiology of cancer (as a complex system) is insufficient, despite the considerable expansion of our knowledge of its genetic and molecular mechanisms (reductionist approach). As mentioned above, new approaches consist of modular treatments that consider processes associated with cancer (such as inflammation, coagulation, etc.) or even forms of eco-systemic treatment that address the entire functioning of the cancer ecosystem (i.e., the microenvironment in which the tumor arises and develops). Finally, without denying the involvement of the genetic–informational component in cancer, this theory invites a balance shift, from a totally gene-centered approach to cancer towards an approach emphasizing the integrated cellular-to-tissue or tissue-to-cellular features of the disease. The theory I propose, therefore, represents a new interpretive model that could help to better understand the pathogenesis of cancer as well as the pharmacological bases for new therapeutic approaches to cancer.

## Abbreviations

SETOC	SETOC: systemic–evolutionary theory of cancer;
DNA	deoxyribonucleic acid
mtDNA	mitochondrial DNA
